# A Comparative Analysis of Endoscopic Carpal Tunnel Release Performed Under Wide-Awake, Local Anesthesia, No Tourniquet in an Office-based Procedure Room Versus Operating Room Setting

**DOI:** 10.1016/j.jhsg.2024.09.007

**Published:** 2024-11-01

**Authors:** Madison Milhoan, Winston Scambler, William F. Pientka

**Affiliations:** ∗ Department of Orthopaedic Surgery, JPS Health Network, Fort Worth, TX; † Texas Christian University Burnett School of Medicine, Fort Worth, TX

**Keywords:** Endoscopic, Outcomes, Procedure room, WALANT

## Abstract

**Purpose:**

Wide-awake, local anesthesia, no tourniquet (WALANT) techniques represent a notable advancement in hand surgery by reducing costs and enhancing patient satisfaction. This study aims to compare Disabilities of the Arm, Shoulder, and Hand (DASH) and pain score improvements in patients undergoing endoscopic carpal tunnel release (ECTR) in an office setting under WALANT versus those performed in the operating room under general or regional anesthesia.

**Methods:**

We conducted a retrospective chart review of all patients aged ≥18 years who underwent ECTR during the period November 2020 to December 2022 by a single hand surgeon at a single level-1 trauma center. A total of 286 procedures in 229 patients were included. We recorded patient demographics, procedure setting, preoperative and postoperative outcome scores, DASH scores, visual analog pain scores, and follow-up duration.

**Results:**

Average follow-up was 6.8 weeks. One hundred and twenty-four in-office WALANT procedures and 162 in-operating room procedures were included. Patients undergoing in-office procedures were significantly older (average age of 58 vs 53 years) (*P* = .004). A significant sex difference was noted between the groups, with more women undergoing in-office (*P* < .00001). There was no difference in preoperative pain or DASH scores between groups or in postoperative DASH score improvement; however, postoperative pain scores were significantly lower at 6 weeks in the in-office WALANT cohort (*P* < .00001).

**Conclusions:**

In-office WALANT ECTR shows similar improvements in DASH scores compared with operating room–based procedures, irrespective of anesthesia type. Postoperative pain was significantly (*P* < .00001) less in the WALANT cohort at 6 weeks. Widespread adoption of office-based WALANT ECTR release could offer substantial financial benefits to both patients and the health care system at large, without compromising patient outcomes.

**Type of study/level of evidence:**

Therapeutic IV.

Wide-awake local anesthesia/no tourniquet (WALANT) procedures in an office-based procedure room offer several advantages over the traditional operating room setting. The primary advantage of WALANT procedures is the reduction in complications associated with general anesthesia while simultaneously allowing for real-time feedback to the surgeon, reducing the risk of nerve damage or injury to surrounding structures,^1^^,^^2^ and allowing for intraoperative assessment of procedure effectiveness (elimination of triggering, flexor tendon gapping, etc). Additionally, conducting these procedures in a clinic setting is often more convenient for patients and can be more cost effective for health care facilities by avoiding operating room costs, including those related to personnel and equipment.^3^ A meta-analysis projected that adopting WALANT for carpal tunnel release (CTR) alone could save $2.3 billion in health care costs over the next decade.^4^ These benefits could contribute to better overall outcomes in the surgical management of carpal tunnel syndrome, a condition affecting approximately 3% of the US population annually.

Although WALANT techniques are well-established internationally, their use remains less prevalent in the United States. Demonstrating comparable or superior efficacy of in-office hand surgery is crucial for justifying and supporting the broader adoption of this technique in the United States, potentially reducing overall health care costs without compromising patient outcomes. Wide-awake, local anesthesia, no tourniquet procedures provide great patient satisfaction, with a documented reduction in patient anxiety and improved overall patient satisfaction.^5^

Comparative studies have shown that endoscopic CTR (ECTR) and open CTR provide similar symptom relief and Boston Carpal Tunnel Questionnaire scores. Endoscopic CTR has been associated with better early recovery of grip and pinch strength and reduced scar tenderness, although no significant clinical advantage has been observed beyond 6 months.^6^ Despite similar long-term outcomes, patients generally prefer endoscopic techniques.^7^

This study aims to evaluate patient-reported outcomes following WALANT ECTR performed in an office-based setting. These outcomes will be compared with those of the same procedure conducted in an operating room. We hypothesize that WALANT ECTR performed in a less resource-intensive environment (office-based) is equivalent to or better than the procedure performed in a more resource-intensive environment (operating room–based) in terms of Disabilities of the Arm, Shoulder, and Hand (DASH) score improvement and postoperative pain.

## Materials and Methods

Following institutional review board approval, we conducted a retrospective chart review of all adult patients (≥18 years) who underwent single-portal ECTR between November 2020 and December 2022 with a single fellowship-trained hand surgeon. Inclusion criteria include all patients aged ≥18 years who underwent ECTR with the senior author during the study period, with preoperative and at least one postoperative DASH score available for review. Patients younger than 18 years, patients undergoing open CTR, or patients without preoperative and/or at least one postoperative DASH score were excluded. Variables recorded included patient demographics, procedure setting (office vs operating room), type of anesthesia (general or regional for operating room, local for office), occurrence of additional procedures performed concurrently, preoperative and postoperative DASH scores, preoperative and postoperative pain scores, and follow-up duration.

As part of the informed consent process conducted by the senior author, patients were provided with information regarding the option of undergoing their procedure under WALANT in an office-based setting or in the operating room. The choice of anesthesia and surgical setting was determined by the patient.

In-office WALANT procedures were performed in a standard examination room used for minor procedures 1 day a week (Fig.). The room undergoes a terminal clean the night before procedure days. No antibiotics are provided for in-office procedures. For ECTR procedures, 18 mL of 1% lidocaine with 1:100,000 epinephrine mixed with 2 mL of sodium bicarbonate is used. Seven milliliters are injected in the volar distal forearm 5–6 cm proximal to the wrist crease, 3 mL is injected at the incision site, and 10 mL is injected over the carpal tunnel in a retrograde fashion from an insertion point overlying the distal edge of the transverse carpal ligament. Procedures are performed with sterile gloves only after sterile draping of the surgical field. The MicroAire endoscopic soft tissue release system was used for all ECTR procedures through a 1 cm incision at the volar wrist, regardless of procedure location.

**Figure:**
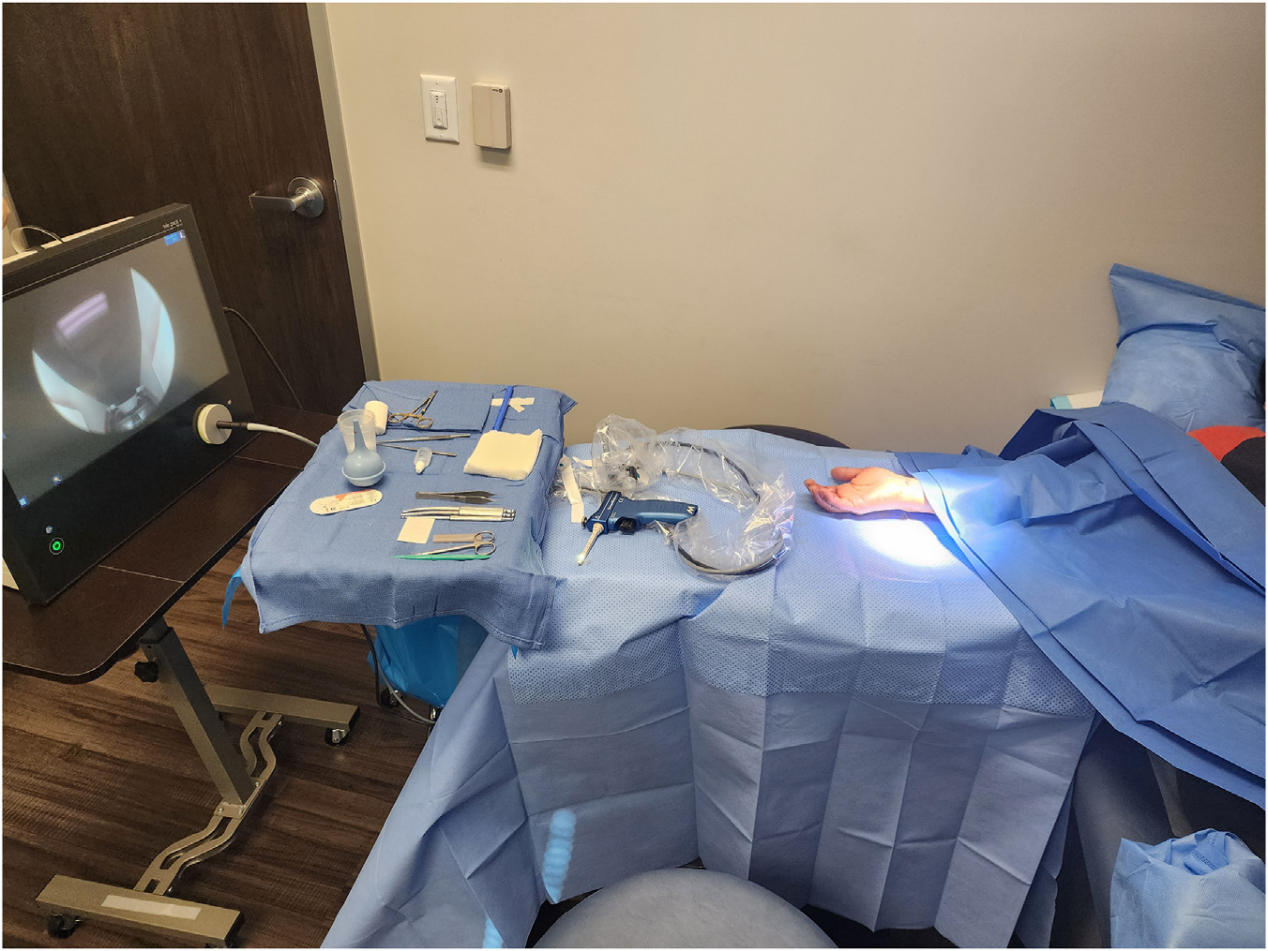
Photograph of in-office procedure room setup for endoscopic carpal tunnel release.

DASH scores are routinely collected immediately before surgery and at the 2- and 6-week postoperative visits. A third visit (and third postoperative DASH score) is scheduled at 12 weeks after surgery if deemed necessary by the senior author at the 6-week after surgery visit based on patient recovery.

Data analysis involved descriptive statistics for numerical data. Independent *t* tests compared age, DASH scores, and postoperative pain scores between the in-office and operating room groups. Chi-square tests assessed sex (biologic) differences, and linear regression analyzed the impact of preoperative DASH scores on postoperative improvement. An a priori power analysis determined a required sample size of 50 per group to detect a minimal 10-point improvement in DASH scores with 80% power, based on an average DASH score of 41 and a standard deviation of 20.^8^

## Results

A total of 318 CTR procedures were initially identified. Patients without at least one preoperative DASH score and one postoperative DASH score recorded were excluded, leaving a total of 286 procedures included in the final analysis. Fifty-seven patients received staged bilateral procedures during the study period, and each procedure was included individually. The final analysis included 124 in-office WALANT procedures and 162 in-operating room procedures. Ninety-three of 162 patients had procedures performed in an ambulatory surgery center (ASC), whereas 69 of 162 were performed in a hospital outpatient surgical department (HOPD). One hundred and forty-two in-operating room procedures were performed under general anesthesia, whereas 20 were performed under regional anesthesia.

Compared with in-office versus in-operating room procedures, the average age of in-office was 57.4 years versus 52.7 years in-operating room (*P* = .004). A statistically significant difference was found in sex between the two groups, with more women undergoing in-office (*P* = < .00001). There was no statistically significant difference in DASH improvement after surgery between the groups (*P* = 0.131). There was also no statistically significant difference in preoperative visual analog pain scores or DASH scores between the groups (*P* = .151 and *P* = .062, respectively), although there was a trend toward lower preoperative DASH scores in the operating room group. Postoperative pain scores were less at 2 weeks (*P* = .057) and 6 weeks (< .00001) in the in-office cohort. Average length of follow-up was significantly longer in patients undergoing procedures in the operating room (6.5 vs 7.8 weeks) (*P* = .025). A full depiction of outcomes may be found in Table 1. Linear regression analysis showed no significant impact of preoperative DASH scores on postoperative improvements. No infections or other wound complications were noted in either cohort.

**Table:** Comparison of ECTR Performed In-operating Room Setting Versus Office-based Procedure Room Under WALANT

Variable	In-Office Procedure (Range)	In-Operating Room Procedure (Range)	*P* Value
Age (y)	57.54 (32–76)	52.71 (23–87)	*.004*∗
Sex (M/F)	20/106	69/27	*< .00001*∗
Preoperative DASH	48.32 (12.5–90.83)	52.48 (0.83–91.67)	.062
DASH improvement	21.5 (−25.84 to 60.00)	19.21 (−38.34 to 71.67)	.131
Preoperative pain score	4.43 (0–10)	4.02 (0–10)	.151
2-wk postop pain score	2.60	3.31	.057
6-wk postop pain score	2.14	4.12	*< .00001*∗
No. of follow-up visits	1.84 (1–4)	1.92 (1–4)	.084
Length of follow-up (weeks)	6.55 (2–32)	7.82 (2–40)	*.025*∗

∗ Statistically significant values.

## Discussion

Our study aimed to compare patient-reported outcomes of ECTR performed using WALANT in an office-based setting versus the traditional operating room setting under general or regional anesthesia. Consistent with existing literature, we found no significant differences in postoperative DASH scores, between the two settings.^9^, ^10^, ^11^ However, we did find that in-office WALANT ECTR had significantly lower postoperative pain compared with procedures performed in the operating room at 6 weeks postprocedure (*P* < .00001). The equivalent improvement in DASH scores in both settings, even after controlling for preoperative scores and pain levels, supports the notion that WALANT is a viable alternative to operating room–based procedures. This aligns with previous findings suggesting that WALANT techniques can provide comparable (or improved) efficacy at a lower cost.^4^

Research trends surrounding WALANT techniques versus traditional operating room–based procedures reveal a shift toward optimizing patient outcomes and procedural efficiency. Studies increasingly highlight the benefits of WALANT in outpatient settings, emphasizing its role in reducing anesthesia-related complications, lowering overall costs, and improving patient satisfaction. For instance, investigations have shown that patients undergoing WALANT procedures often experience shorter recovery times and less immediate postoperative pain compared with those receiving general anesthesia in traditional operating room settings.^1^ This trend aligns with broader health care initiatives aimed at enhancing patient-centered care and reducing hospital stays.^12^

Moreover, ongoing research is examining the versatility of WALANT across various surgical specialties beyond hand surgery. As surgeons gain more experience with WALANT, studies are exploring its application for complex procedures, assessing not only the technical feasibility but also long-term outcomes and complications. The growing body of evidence suggests that WALANT could serve as a model for transitioning other surgical disciplines toward more efficient and less invasive practices. Overall, the emphasis on patient experience and streamlined surgical workflows continues to drive interest in WALANT, setting a precedent for the future of hand surgical care.^1^^,^^13^

When analyzing patient demographics between the two groups in this study, we interestingly found that patients in the in-office group were more likely to be women and more likely to be older. This result has been echoed in another study evaluating WALANT versus sedation for CTR that demonstrated younger patients choosing the operating room over the office.^14^ One possible explanation for this finding is both a provider and patient preference for avoidance of general anesthesia in the setting of multiple medical comorbidities. Given the increasing prevalence of additional medical diagnoses with age, WALANT offers a safer alternative for the management of carpal tunnel syndrome in these patients in particular. To our knowledge, no prior study has demonstrated a sex difference between patients electing to proceed with CTR in the operating room versus in an office-based setting. Prior work has emphasized trust being a key factor driving patient’s preferred anesthesia type.^15^ Further studies evaluating concerns about wide-awake procedures between sexes may help shed light on this issue, especially considering our finding that women were more likely to elect for an in-office endoscopic CTR.

Although our specific study did not assess costs between the two groups, there is a demonstrated notable reduction in the financial impact of a CTR performed in-office through cost savings during each phase of care, including the procedure and the immediate postoperative period.^4^ The professional fee portion of the procedure remains consistent regardless of the procedure setting, but the decreased costs of in-office CTR result from the elimination of anesthesia and hospital/operating room charges. Previous reports have estimated general/regional anesthesia costs for carpal tunnel procedures to be $654 more than local only, and facility charges for ASCs averaged $2,309, whereas HOPD procedure charges averaged $2,868.^16^^,^^17^ Based on these reports, in-office CTR can eliminate between $2,963 (ASC) and $3,518 (HOPD) per CTR (approximately 80% of the total cost of care related with ECTR). It must be noted, however, that there are some costs to establishing an in-office procedure program stemming from instrumentation costs, sterilization of instruments, and disposables such as surgical drapes, gloves, and dressing supplies. Prior work has also demonstrated decreased procedural, as well as door-to-door times, for the WALANT technique for ECTR.^14^ Wide-awake, local anesthesia, no tourniquet ECTR performed in the office can be performed more efficiently because of decreased room turnover and preoperative assessment requirements, which decrease patient costs while simultaneously effectively increasing profitability for the surgeon by decreasing case duration. In light of touted similar outcomes, the WALANT procedure offers considerable benefits to both the patient and the health care system.

There are a few limitations to our study. This study includes outcomes from a single surgeon at a single institution. Therefore, these results may affect generalizability. We also have a total mean follow-up of 6.8 weeks, which may not capture long-term outcomes and complications. Furthermore, we did not collect or analyze patient comorbidities, which could have a potential impact on patient outcomes. The length of follow-up between cohorts is significantly different; however, the number of follow-up visits demonstrated no difference. This finding suggests that outliers in the in-operating room cohort increased the average follow-up without additional postoperative visits, presumably from patient-initiated postponement/rescheduling of final postoperative visits.

Overall, we find office-based WALANT ECTR to be reliable with similar patient-reported outcomes compared with the traditional ECTR performed in the operating room. We believe that widespread adoption could lead to notable financial and physical benefits to both the patient and health care system at large.

## Conflicts of Interest

The senior author (W.P.) is a consultant for MicroAire but received no external support or funding for this project.
